# Snus, nicotine pouches and cardiovascular disease: Evidence, mechanisms, and the challenge of novel nicotine products

**DOI:** 10.1111/joim.70160

**Published:** 2026-09-07

**Authors:** Magnus Olsson, Hanne Tønnesen, Jakob Hedberg, Johan Sundström, Eva Nordendahl, Björn Klinge, Michael Fored, Cecilia Magnusson, Daniel Jönsson, Peter Persson, Aron Naimi‐Akbar

**Affiliations:** ^1^ Faculty of Odontology, Health Technology Assessment‐Odontology (HTA‐O) Malmö University Malmö Sweden; ^2^ WHO‐CC for Evidence‐Based Clinical Health Promotion, The Parker Institute, Bispebjerg‐Frederiksberg Hospital University of Copenhagen Frederiksberg Denmark; ^3^ Department of Surgical Sciences Uppsala University Uppsala Sweden; ^4^ Department of Medical Sciences, Clinical Epidemiology Uppsala University Uppsala Sweden; ^5^ Clinical Epidemiology Division, Department of Medicine Karolinska Institutet Solna Sweden; ^6^ Department of Periodontology Eastman Institute, Stockholm AB Stockholm Sweden; ^7^ Department of Dental Medicine Periodontology and Dental Prophylaxis Unit Karolinska Institutet Huddinge Sweden; ^8^ Department of Global Public Health Karolinska Institutet Solna Sweden; ^9^ Centre for Epidemiology and Community Medicine Stockholm Health Care District Stockholm Sweden; ^10^ Department of Clinical Sciences Malmö Lund University Malmö Sweden; ^11^ Public Dental Service of Skåne Lund Sweden; ^12^ Division of Oral Diagnostics and Surgery, Department of Dental Medicine Karolinska Institutet Huddinge Sweden

**Keywords:** cardiovascular disease, harm reduction, nicotine pouches, nicotine, smokeless tobacco, snus, SWETOC, tobacco‐specific nitrosamines

## Abstract

Conventional Swedish snus is used by millions in Scandinavia and increasingly worldwide, yet its cardiovascular effects remain poorly characterized. The emergence of nicotine pouches—commonly referred to as ‘white snus’ in Sweden—that deliver comparable nicotine without tobacco constituents adds complexity to a heterogeneous landscape and raises questions about whether cardiovascular risk follows nicotine exposure regardless of product type. We explore epidemiological and mechanistic evidence on snus and cardiovascular disease, the contributions of nicotine versus tobacco‐specific nitrosamines (TSNAs) to risks, and identify knowledge gaps, with focus on novel nicotine products and the research opportunity offered by the Swedish Tobacco Cohort (SWETOC). Epidemiological evidence suggests elevated cardiovascular disease (CVD) mortality among snus users, with the most consistent signals for fatal myocardial infarction and heart failure. Associations with incident atherosclerotic events are more heterogeneous. Mechanistic evidence points to nicotine‐mediated pathways, including sympathetic activation, endothelial dysfunction and increased arterial stiffness, whereas evidence for TSNA‐driven CVD risk remains sparse, and declining TSNA levels in modern Swedish snus make that path less likely and harder to study. Nicotine pouches deliver comparable or higher systemic nicotine than conventional snus but lack long‐term cardiovascular outcome data. Real‐world cessation data from Denmark suggest structured programmes can achieve 6‐month abstinence rates of approximately 53% among non‐smoking nicotine users. The evidence supports nicotine as the primary driver of snus‐associated CVD risk, with important implications for the cardiovascular safety of novel nicotine products. Evidence is needed to inform public health guidance, clinical advice and regulatory decisions. SWETOC represents a unique infrastructure to generate this evidence.

## Graphical abstract

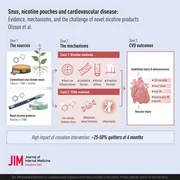

## Introduction

Cardiovascular disease (CVD) remains the leading cause of death worldwide, and tobacco use is among its most important modifiable risk factors. In Sweden, CVD accounted for about 32% of all deaths in 2023, approximately 30,000 deaths annually [1]. The global burden of disease shows that an estimated 19.4 million deaths were attributed to CVD globally in 2021, representing a 57.5% increase in absolute numbers since 1990. Despite this rise, age‐standardized death rates have declined by 34.3% over the same period, reflecting improvements in prevention and treatment, predominantly in high‐income countries [2]. Although age‐standardized CVD mortality has declined substantially over recent decades, largely attributable to improved treatment and reductions in smoking, the burden remains considerable. Sweden has the lowest smoking prevalence in the European Union [3], partly attributed to the widespread use of snus as a cigarette substitute, making the Swedish experience a natural experiment of considerable public health relevance. Although the harms of cigarette smoking are well established, the cardiovascular risk profile of smokeless tobacco, and Swedish snus in particular, is considerably less clear. Snus is a moist, pasteurized oral tobacco product that has been used in Sweden for centuries and has expanded into other Scandinavian countries and, more recently, North America.

Throughout this review, we use the term snus to refer to the traditional tobacco‐based Swedish moist oral tobacco product, sometimes referred to as brown snus or Swedish snus in other contexts. Tobacco‐free nicotine pouches, commonly referred to as white snus in Sweden since their introduction in 2016, are treated as a distinct product category and referred to as nicotine pouches throughout. We note that the lack of consistent terminology in both the scientific literature and public discourse complicates comparisons across studies and may contribute to confusion among consumers and policymakers regarding the differing risk profiles of these products.

Snus is used by placing a portion under the upper lip, where it remains in contact with the oral mucosa for extended periods, typically several hours per day [4]. Each portion delivers nicotine through the buccal mucosa in a slower and more sustained manner than inhalation. Unlike fermented smokeless tobacco products used in South Asia (gutka, khaini and paan), snus is manufactured by pasteurization rather than fermentation, resulting in relatively low levels of tobacco‐specific nitrosamines (TSNAs) [5]. Snus has been subject to quality standards since the introduction of the GothiaTek standard, an industry initiative originally developed by Swedish Match [5], and TSNA levels are also regulated by the Swedish Food Safety Authority [6]. Importantly, TSNA levels have declined substantially over several decades, meaning that older epidemiological studies were conducted at a time when constituent levels were considerably higher than today [6]. This product‐specific feature is central to interpreting the literature: Studies on Asian or American smokeless tobacco products, which are typically fermented and may contain higher levels of certain toxicants, are not directly generalizable to snus.

Nicotine pouches are placed under the lip in the same manner as snus but contain no tobacco leaf, using instead a cellulose‐based matrix with added nicotine, flavourings and sweeteners. Although labelled as tobacco‐free, the nicotine in most commercial products is in practice derived from tobacco plants [7, 8]. Nicotine is added separately, and as a result the nicotine content varies considerably across brands and variants, from approximately 2 to 19 mg per portion in Swedish products, with many popular variants delivering higher nicotine doses than snus [7, 9]. The pH of nicotine pouches is adjusted to alkaline levels, with alkaline pH values observed in virtually all commercial products examined, resulting in a high proportion of free‐base nicotine and facilitating mucosal absorption, a feature not inherent to snus [10]. These products are now widespread among younger users across the Nordic countries and North America, and their use has grown rapidly since their introduction [7, 11, 12].

The prevalence of daily snus use in Sweden has remained relatively stable among men at approximately 18%–22% over the past two decades, whereas use among women has increased substantially, reaching approximately 10% in 2024 (Fig. 1) [3]. The increase is particularly pronounced among young women aged 16–29 years, among whom daily snus use has increased more than sixfold since 2018, from approximately 3% to 18% in 2024 [3]. Note that from 2022, the Swedish national health survey combines snus and nicotine pouches under a single measure, meaning that recent figures reflect use of both product types. Daily use of nicotine pouches specifically has increased sharply among young adults aged 16–29 years, with prevalence rising from approximately 10% to 15% among women and from 5% to 11% among men between 2022 and 2024 (Fig. 2) [3]. These trends underscore the rapidly changing nicotine landscape in Sweden and the growing public health relevance of understanding the cardiovascular effects of both snus and nicotine pouches.

**Fig. 1:**
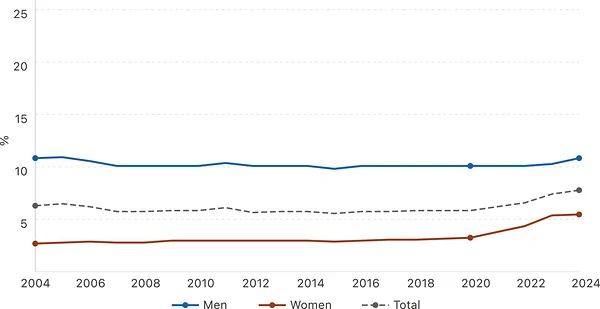
Prevalence (%) of daily snus use among people aged 16–84 in Sweden, 2004–2024, by sex; adapted from data from the National Public Health Survey 2024 [3].

**Fig. 2:**
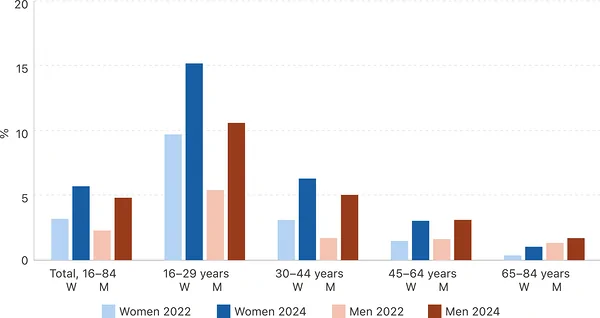
Prevalence (%) of daily nicotine pouch use in Sweden, 2022 and 2024, by sex and age group; based on data from the National Public Health Survey 2024 [3].

E‐cigarette use in Sweden remains relatively low overall but has increased substantially in recent years, particularly among young people. In 2024, approximately 4% of the Swedish population aged 16–84 reported using e‐cigarettes, up from 1.3% in 2020 [3]. The increase is most pronounced among young adults aged 16–29 years, where prevalence rose from 2% in 2020 to 13% among women and 7% among men in 2024 [3]. Although e‐cigarette prevalence remains higher among young women than young men, use rose from 2% to 7% among men and from 2% to 13% among women over this period, representing more than a tripling among men and a sixfold increase among women [3]. Internationally, recruitment to e‐cigarette use occurs predominantly among adolescents and young adults, whereas direct cardiovascular outcome data in this age group remain very limited, making the rising prevalence among younger users particularly relevant from a preventive CVD perspective [13].

The cardiovascular effects of e‐cigarettes are an area of active investigation, and the evidence base remains incomplete. Experimental studies indicate that acute e‐cigarette inhalation with nicotine has detrimental effects on vascular function. A randomized crossover study conducted in healthy volunteers showed that inhalation of 30 puffs of nicotine‐containing e‐cigarette aerosol caused increased platelet and fibrin‐dependent thrombus formation and impaired microvascular dilatation capacity, with effects observed within 15 min of exposure and no comparable effects seen with nicotine‐free aerosol, indicating nicotine as the primary mediator [14]. The same research group previously demonstrated that nicotine‐containing e‐cigarette vapour increased circulating endothelial and platelet‐derived extracellular vesicles, markers of vascular injury and activation [15]. In a systematic review and meta‐analysis of randomized trials, nicotine‐containing e‐cigarettes acutely increased arterial stiffness—measured as pulse wave velocity and augmentation index—compared with nicotine‐free e‐cigarettes, supporting nicotine as an important driver of acute vascular effects [16]. The most consistent human signal across studies relates to sympathetic nervous system activation, including heart rate variability changes and blood pressure and heart rate responses, with nicotine identified as a key mediator in a systematic review of autonomic cardiovascular effects [17]. A systematic review and meta‐analysis found limited evidence for an association between e‐cigarette use and clinical CVD or cardiac dysfunction, though the interpretation is substantially complicated by heterogeneity across device generations, nicotine formulations and liquid compositions, which has been highlighted as a barrier to risk quantification and an argument for clearer product standards and surveillance [18].

In Sweden, e‐cigarettes and nicotine‐containing refill liquids are regulated under the Tobacco and Similar Products Act, whereas tobacco‐free nicotine products, such as nicotine pouches, are regulated under a separate act introduced in January 2023 [19]. Both acts include provisions on marketing, labelling and sales, including an age limit of 18 years. Notably, the nicotine content of nicotine pouches remains unregulated under current Swedish legislation.

The cardiovascular profile of nicotine pouches is largely unstudied in long‐term outcome data, but they provide a unique mechanistic opportunity to test two competing hypotheses about the drivers of snus‐associated CVD risk. The first holds that risk is mediated primarily by nicotine, through haemodynamic and endothelial mechanisms, and would therefore predict similar cardiovascular risk from nicotine pouches despite the absence of tobacco. The second holds that tobacco‐specific constituents, including TSNAs, contribute independently to CVD risk, in which case nicotine pouches might carry a more favourable cardiovascular profile. This review synthesises current epidemiological and mechanistic evidence on snus and CVD, evaluates the relative support for each hypothesis, and delineates the knowledge gaps that must be addressed, particularly in light of novel nicotine products.

## Epidemiological evidence

### Mortality and fatal cardiovascular events

The most consistent epidemiological signal for snus and CVD is an elevated risk of CVD mortality, though findings are not uniform across all study populations. Meta‐analyses of global smokeless tobacco literature suggest stronger relative risks for fatal than non‐fatal cardiovascular outcomes. Pooled estimates indicate relative risks of 1.13 for fatal myocardial infarction and 1.40 for fatal stroke among ever users, with excess risk restricted to current users [20]. A later meta‐analysis corroborated these findings, reporting relative risks of 1.15 for fatal ischaemic heart disease and 1.39 for fatal stroke, while finding no significant increase in non‐fatal outcomes overall, with the association with non‐fatal ischaemic heart disease significant in Asian but not European studies [21]. A third meta‐analysis found no significant overall association between smokeless tobacco use and coronary heart disease but reported a relative risk of 1.37 for fatal coronary heart disease specifically among snus and snuff users in the European region, with an attributable fraction of 5% for fatal coronary heart disease in Europe [22]. Taken together, these meta‐analyses consistently point to a pattern where smokeless tobacco use is more strongly associated with fatal than non‐fatal cardiovascular outcomes, and where European snus users show a distinct risk profile compared with users of other smokeless tobacco products. Findings from Swedish‐specific data are more heterogeneous, with some analyses reporting attenuated or null associations with CVD mortality after adjustment for confounders [23]. This pattern, where snus appears to affect the severity and acute course of cardiovascular events more than their incidence, is an important and underappreciated feature of the literature.

### Myocardial infarction

A pooled analysis of AMI incidence among never‐smoking men found no statistically significant increase with current snus use (HR 1.04; 95% confidence interval [CI] 0.93–1.17), while suggesting a possible elevation in 28‐day case fatality (HR 1.28; 95% CI 0.99–1.68), with the excess mortality concentrated within the first 24 h after the event [24]. This pattern is consistent with haemodynamic and endothelial mechanisms that may increase susceptibility to event lethality rather than atherosclerotic plaque progression per se. Acute snus use produces sympathetically mediated increases in heart rate and blood pressure [25, 26], and chronic use is associated with endothelial dysfunction, impaired nitro‐glycerine response, and increased arterial stiffness [27], a vascular phenotype that may worsen myocardial perfusion and haemodynamic stability during an acute ischaemic event. Sustained sympathetic activation from habitual snus use may also increase myocardial oxygen demand, and catecholamine release may lower the threshold for arrhythmias under proarrhythmic conditions, such as myocardial ischaemia [26, 28]. Snus cessation post‐MI was associated with an approximately halved mortality risk compared with continued use (HR 0.51; 95% CI 0.29–0.91) [29], and continued snus use after percutaneous coronary intervention (PCI) was associated with shorter time to repeat PCI [30].

### Stroke

Pooled analyses among men without a smoking history show no statistically significant association between snus use and stroke incidence, with a hazard ratio (HR) of 1.04 and a 95% CI of 0.92–1.17, but suggest increased post‐stroke mortality, HR 1.32 (95% CI 1.08–1.61) [31]. A subsequent cohort study reported a statistically significant increased risk of total and ischaemic stroke among never‐smokers, with HRs of 1.52 and 1.63, respectively [32]. These findings should be interpreted with caution, however, as they derive from a single cohort of older adults with relatively wide CIs and limited statistical power for stroke outcomes. A more recent cohort study with 27 years of follow‐up in non‐smoking middle‐aged men found no significant elevation in stroke risk [33]. Taken together, the available evidence does not support a consistent causal link between snus use and stroke, though a modest effect cannot be excluded.

### Heart failure

Heart failure appears to be among the more consistent positive epidemiological signals for snus in Swedish data. In two independent Swedish cohorts, current snus use was associated with increased heart failure risk. In the Uppsala Longitudinal Study of Adult Men, an older community‐based cohort, the HR for heart failure overall was 2.08 (95% CI 1.03–4.22), and 2.55 for non‐ischaemic heart failure. In the larger Construction Workers Cohort of never‐smoking men, the corresponding HR was 1.28 (95% CI 1.00–1.64) [23]. A more recent cohort study with 27 years of follow‐up reported an HR of 1.54 (95% CI 1.12–2.11) for heart failure [33]. A systematic review and meta‐analysis found no clear increase in ischaemic heart disease among Swedish snus users, in contrast to US smokeless tobacco users, suggesting that heart failure and hypertension emerge more consistently than ischaemic incidence in Swedish data [34]. This pattern is consistent with a haemodynamic mechanism: acute snus use produces sympathetically mediated increases in heart rate and blood pressure [35], and chronic use has been associated with impaired endothelial function and increased arterial stiffness [27, 36, 37], providing a plausible biological pathway to heart failure development.

### Atrial fibrillation and hypertension

A pooled analysis of seven prospective Swedish cohort studies found no increased risk of atrial fibrillation among current snus users (RR 1.07; 95% CI 0.97–1.19) [38]. For hypertension, a large prospective study of never‐smoking men found a moderately elevated risk among snus users, with RR 1.39 for incident high blood pressure and RR 1.36 for registered hypertension [39], and a more recent cohort study with 27 years of follow‐up reported an HR of 1.33 for hypertension [33]. However, not all analyses have found consistent associations with incident hypertension, and a systematic review found no clear increase among Swedish snus users in particular [34], warranting cautious causal interpretation.

### Evidence from national health authority assessments

Independent assessments by national public health bodies in Scandinavia broadly corroborate the epidemiological signals described above. The Norwegian Institute of Public Health, commissioned by the Ministry of Health and Care Services, conducted a systematic evaluation of health risks from snus use and concluded that snus probably increases the risk of hypertension and lethality after myocardial infarction and stroke; that cessation of snus after a heart attack may halve the risk of dying during the subsequent 2 years; that snus possibly increases the risk of endothelial dysfunction and has immediate effects on cardiac function, including reduced diastolic function; and that uncertainty remains regarding associations with incident myocardial infarction, stroke, atrial fibrillation and chronic heart failure [40].

Cross‐sectional data from the Norwegian HUNT3 study found that extensive snus use was associated with higher systolic blood pressure and larger waist circumference compared with non‐users after adjustment for major confounders, though HDL‐cholesterol was also higher among snus users [25]. This mixed cardiometabolic profile underlines the complexity of risk assessment and the difficulty of summarizing snus‐associated CVD risk in simple terms.

## Biological mechanisms: nicotine versus tobacco constituents

Two broad mechanistic hypotheses can be formulated to explain snus‐associated CVD risk, and their relative importance has direct implications for the cardiovascular profile of novel nicotine products.

### Track 1: Nicotine‐mediated pathways

Nicotine acts on nicotinic acetylcholine receptors in sympathetic ganglia and the adrenal medulla, triggering release of noradrenaline and adrenaline. Acute snus use raises blood pressure and heart rate and produces signs of sympathetic activation and peripheral vasoconstriction [35, 41]. Whether these haemodynamic effects attenuate with chronic daily exposure due to tolerance development remains unclear, as longitudinal data specifically examining habituation of blood pressure responses in snus users are lacking.

Chronic snus use has been associated with impaired endothelial function, demonstrated by reduced flow‐mediated dilation in experimental studies [36] and a tendency towards reduced flow‐mediated dilation in a large population‐based cross‐sectional sample [27], as well as increased arterial stiffness [37]. These vascular changes provide a plausible biological pathway to heart failure development and increased post‐event mortality.

Nicotine may also contribute to insulin resistance and an adverse lipid profile, and catecholamine release may lower the threshold for arrhythmias under proarrhythmic conditions such as myocardial ischaemia [26]. The evidence base for these mechanisms in snus users specifically is limited, and the claims above draw primarily on general nicotine pharmacology.

Supporting the primacy of nicotine as a cardiovascular toxicant, prenatal nicotine exposure through maternal snus use, without concurrent smoking, was associated with elevated systolic blood pressure and altered heart rate variability in preschool children, providing a rare opportunity to observe nicotine's cardiovascular effects in isolation from combustion products [42].

Whether snus use promotes platelet activation or a prothrombotic state remains insufficiently studied in snus‐specific data, and no robust conclusions can currently be drawn from available evidence. This suggests that snus‐associated CVD mortality may be driven primarily by haemodynamic and endothelial mechanisms, though the indirect proatherogenic environment created by chronic blood pressure elevation and endothelial dysfunction may still promote thrombosis over time.

Sex differences in the cardiovascular response to snus represent an underexplored but potentially important dimension. Acute snus exposure has been shown to elicit divergent haemodynamic responses in men and women, with significant increases in blood pressure and heart rate observed in women but not in men [41]. Among young Swedish adults, snus use was associated with sex‐specific cardiometabolic effects, including higher BMI and waist circumference in women [43].

### Track 2: Tobacco‐specific nitrosamine‐mediated pathways

TSNAs—primarily nicotine‐derived nitrosamine ketone and *N*‐nitrosonornicotine—are potent carcinogens formed during tobacco processing and well‐established causes of cancer of the lung, pancreas and oral cavity. Beyond their carcinogenic effects, TSNAs and their metabolites have been shown to cause cellular injury through disruption of intracellular signalling, formation of DNA and protein adducts, and promotion of oxidative stress and inflammation [44]. These mechanisms could in principle contribute to endothelial dysfunction and a prothrombotic state, though direct human data linking TSNA exposure specifically to CVD outcomes in snus users are lacking. The declining TSNA content of modern Swedish snus, driven by the GothiaTek standard and regulatory limits set by the Swedish Food Safety Authority, substantially weakens the case for a TSNA‐driven explanation of contemporary CVD risk [5, 6]. Older epidemiological studies were conducted at a time when TSNA levels were considerably higher, complicating causal inference from the available data. Importantly, the assumption that nicotine pouches are entirely free of TSNAs requires qualification. A chemical analysis of 44 commercial nicotine pouch products detected TSNAs in 26, with levels reaching up to 13 ng *N*‐nitrosonornicotine per pouch [10]. Although these levels are substantially lower than those found in traditional tobacco products, their presence in products marketed as tobacco‐free raises questions about manufacturing processes and quality control and underscores the need for independent chemical surveillance of novel nicotine products.

## Sex differences in cardiovascular risk from snus and novel nicotine products

Women represent a growing proportion of snus and nicotine pouch users in Sweden, yet they remain conspicuously absent from most of the epidemiological literature on snus and CVD, which has been conducted almost exclusively in men. The Norwegian Institute of Public Health explicitly noted the complete absence of studies examining CVD outcomes from snus use in women [40], and a recent systematic review confirms that evidence on the health effects of novel nicotine products in women remains critically sparse [45].

The available mechanistic data suggest that this gap is not merely a matter of statistical power but may reflect genuine sex differences in the cardiovascular response to nicotine. Acute snus exposure has been shown to elicit divergent haemodynamic responses in men and women, with significant increases in blood pressure and heart rate observed in women but not in men [41]. Among young Swedish adults, snus use was associated with sex‐specific cardiometabolic effects, including higher BMI and waist circumference in women [43]. Cross‐sectional data from the Norwegian HUNT3 study similarly showed that snus use was associated with higher systolic blood pressure and larger waist circumference, a pattern that may be particularly relevant given the rising use among young women [25].

These findings suggest that women who use nicotine products may carry a disproportionate cardiovascular risk relative to their exposure level and underscore the urgent need for sex‐stratified analyses of snus‐ and nicotine pouch‐associated CVD outcomes. As snus and nicotine pouch use continues to rise among young women in Sweden, generating this evidence is no longer optional but a matter of public health urgency.

## Novel nicotine products and critical knowledge gaps

### Nicotine pouches

Nicotine pouches (e.g., ZYN, VELO) have grown rapidly in market share, particularly among younger users. Pharmacokinetic studies show that nicotine delivery from pouches is comparable to, and at higher doses can exceed, that from conventional snus [46]. Short‐term experimental studies demonstrate pronounced heart rate responses at higher nicotine doses: Heart rate increased by approximately 25 bpm with a 30 mg nicotine pouch, comparable to that observed with cigarette smoking (approximately 27 bpm), and arterial stiffness was elevated across all products [47], consistent with the sympathomimetic pathway described above. However, there are no published long‐term cohort studies examining incident CVD or mortality in nicotine pouch users. The short time since widespread adoption is a fundamental limitation, but prospective data collection should be prioritized.

Many popular nicotine variants deliver substantially higher nicotine doses than conventional snus. Although a standard portion of Swedish snus contains approximately 8 mg of nicotine, nicotine products available on the Swedish market typically range from 2 to 19 mg per portion [7], but products available on the broader European market, including via online retail, reach up to approximately 50 mg per portion [10], with high‐strength variants delivering nicotine doses that may substantially exceed those of conventional snus. Importantly, unlike snus, whose nicotine content has remained relatively stable across products, nicotine pouches are marketed in a wide range of strengths, and particularly younger users may preferentially select higher strength products or use multiple portions simultaneously.

If nicotine is indeed the primary driver of snus‐associated CVD risk, the relevant question is not merely whether nicotine pouches carry similar risk to snus, but whether their higher and more variable nicotine delivery could translate into greater cardiovascular harm. The dose–response relationship between chronic nicotine exposure and CVD risk has not been well characterized for any oral nicotine product, and this represents a major evidence gap. The acute haemodynamic effects of nicotine are clearly dose‐dependent [47], but whether this translates into a graded long‐term risk of heart failure, fatal myocardial infarction or cardiovascular mortality is unknown. Given that nicotine pouch use is growing fastest among young adults with decades of potential exposure ahead, and that the nicotine content of these products remains unregulated in Sweden, this knowledge gap has direct regulatory implications.

### Methodological challenges across the literature

Several methodological challenges complicate synthesis of the existing evidence. (1) Exposure classification is inconsistently defined across studies, covering current versus former versus never use, dose and duration, dual use with cigarettes and product era, as snus composition has changed over decades. Within‐person variances in tobacco exposures over time are also unknown, which demands repeated exposure collection. (2) The distinction between incident CVD and case fatality is often not analytically separated, yet these may reflect different mechanisms. (3) Healthy user bias may affect snus cohorts if users who perceive symptoms reduce or stop use prior to events. (4) Time‐varying exposure, particularly cessation after CVD events, creates complex confounding structures that require careful methodological handling. (5) The distinction between short‐term experiments and long‐term habitual use is frequently conflated in policy discussions and deserves explicit clarification. For example, nicotine replacement therapy can be expected to carry cardiovascular risk proportional to the dose and duration of exposure, but the available evidence stems from short trials in healthy populations. (6) The rapid proliferation of new nicotine delivery systems, including nicotine pouches and e‐cigarettes, introduces additional product heterogeneity that current cohort studies are not yet equipped to address. Exposure to these products is rarely captured in register‐based data, and their cardiovascular effects may differ from conventional snus due to differences in nicotine dose, delivery kinetics and product constituents.

### Priority knowledge gaps

The following gaps are identified as highest priority for future research: (1) long‐term cardiovascular outcome data in nicotine pouch users, to test the nicotine‐versus‐TSNA hypothesis in a natural experiment. (2) Mechanistic studies directly comparing snus and nicotine pouches on endothelial function, arterial stiffness and haemodynamics in matched populations. (3) Better characterization of dose–response relationships and threshold effects for nicotine‐mediated CVD risk, relevant both for snus and NRT. (4) Studies addressing the incidence–fatality discordance, particularly the haemodynamic and arrhythmogenic mechanisms that may explain higher CVD fatality without proportionate incidence elevation. (5) CVD outcome data in women, who are conspicuously absent from most snus epidemiology; a recent systematic review confirms that evidence on health effects of novel nicotine products in women remains critically sparse [45].

### Genetics of nicotine dependence and implications for CVD research

A largely unexplored dimension of snus‐associated CVD risk is the role of genetic factors in both nicotine dependence and its downstream health effects. Nicotine dependence is substantially heritable, and genome‐wide association studies (GWAS) have identified loci influencing initiation, intensity of use and cessation success [48]. Genetic variants in pathways related to nicotine metabolism (CYP2B6), serotonin and dopamine signalling and neurobiological reward circuits contribute to individual differences in dependence severity and the difficulty of cessation [48]. From a CVD perspective, these genetic factors are relevant in two ways: They determine the intensity and duration of nicotine exposure, and thus the cumulative cardiovascular burden, and they may independently modulate CVD risk through shared biological pathways. Linking large‐scale population cohorts with genomic data, such as through integration of Swedish Tobacco Cohort (SWETOC) with existing GWAS resources, offers a promising avenue for disentangling genetic susceptibility to dependence from the direct cardiovascular effects of nicotine exposure. Genetic instruments for nicotine metabolism could be used to examine whether genetically determined differences in nicotine exposure intensity are associated with CVD outcomes, offering a complementary Mendelian randomization approach to the causal effects of nicotine itself.

### The role of SWETOC in addressing the evidence gap

The SWETOC is a nationwide cohort of approximately 5.5 million individuals with prospectively collected tobacco exposure data from the Swedish Public Dental Service, linked to 12 national health and social registers [49]. Adult data (≥18 years) form the analytical foundation for health outcome studies, whereas the inclusion of younger age groups enables studies of early‐life nicotine exposure and age of initiation, increasingly relevant as novel nicotine products recruit younger users. SWETOC captures long‐term, real‐world exposure to Swedish snus across multiple product eras and enables large‐scale register‐based analyses of incident CVD, case fatality and post‐event prognosis with high statistical power. As nicotine pouches become increasingly prevalent in the Swedish population, SWETOC is positioned to generate the first long‐term cohort evidence on their cardiovascular effects—directly addressing the central question of whether CVD risk follows nicotine exposure regardless of product type. A planned update of the SWETOC data infrastructure, incorporating additional data collection rounds, may further strengthen the cohort's capacity to capture the cardiovascular effects of newer nicotine products as their users accumulate sufficient follow‐up time, though any such analyses will require careful consideration of the limited follow‐up currently available for nicotine pouch users.

## Perspective

The tobacco industry has positioned smokeless tobacco products, and more recently nicotine pouches, as instruments of harm reduction. The evidence reviewed here suggests that this framing deserves careful scrutiny. Snus use is not without cardiovascular risk and should not be assumed to be a safe alternative to cigarette smoking, given its independent associations with elevated CVD mortality, heart failure risk, and adverse postinfarction prognosis. The mechanistic evidence pointing to nicotine as the primary driver of these effects raises important questions about the cardiovascular safety of nicotine pouches, as products delivering comparable or higher systemic nicotine cannot be assumed to be cardiovascularly neutral on the basis of tobacco constituent removal alone. The growing use of these products among young, never‐smoking populations underscores the importance of generating robust long‐term evidence before drawing firm conclusions about their relative safety.

## Conclusion

Snus use is associated with elevated CVD mortality and, more consistently than atherosclerotic incidence, with heart failure risk and adverse prognosis after cardiac events. The biological mechanism most strongly supported by current evidence is nicotine‐mediated haemodynamic and endothelial dysfunction, rather than tobacco‐specific carcinogens. Reported declines in TSNA content of modern Swedish snus have been cited as weakening the case for a tobacco‐constituent explanation, leaving nicotine as the most plausible explanation for the observed cardiovascular burden [5].

The cardiovascular risk profile of snus differs substantially from that of cigarette smoking. Cigarette smoke contains thousands of combustion‐derived chemicals—including carbon monoxide and polycyclic aromatic hydrocarbons—which contribute to endothelial injury, thrombogenesis and atherosclerosis through pathways independent of nicotine [26, 50, 51]. The substantially higher CVD risk associated with smoking compared with snus, despite comparable systemic nicotine delivery, suggests that combustion products are important additional contributors to smoking‐associated cardiovascular risk (Table 1).

**Table 1:** Nicotine content and delivery characteristics of common nicotine products.

Product	Nicotine content per portion/use	Nicotine absorbed	Route of use
Cigarette	10–13 mg per cigarette	∼1 mg per cigarette	Inhalation of smoke (combustion)
E‐cigarette	Max 20 mg/ml (disposable); self‐regulated with refill liquids [52]	1.2–1.3 mg per 15 puffs	Inhalation of heated vapour
Snus	8–13 mg per portion [53]	∼2.4 mg per portion [46]	Placed under upper lip; oral mucosal absorption
Nicotine pouch	1.8–19 mg per portion (Swedish products); varies widely by brand and variant [10]	∼50% of available nicotine [46]	Placed under upper lip; oral mucosal absorption

*Note*: Adapted from Rosaria Galanti et al. [7]. Nicotine content and absorption figures based on Vedøy and Lund [53], Lunell et al. [46] and Mallock et al. [10]; values may vary across brands and products.

The rapid uptake of nicotine pouches among younger populations, many of whom have no prior tobacco history, raises important questions for public health policy that current evidence cannot fully answer. Whether long‐term use of these products carries meaningful cardiovascular risk remains unknown, and this uncertainty itself argues for caution. Better evidence is urgently needed to inform public health guidance, clinical advice and regulatory decisions. Cohort infrastructure, such as SWETOC, with its capacity to follow millions of individuals across product eras, is positioned to generate precisely this kind of real‐world evidence.

In the meantime, real‐world data from Danish intensive cessation programmes offer cautious grounds for optimism. Among non‐smoking nicotine users enrolled in the Danish STOPbasen programme, approximately 53% achieved continuous nicotine abstinence at 6 months [54]. Notably, 59% of participants used snus, nicotine pouches or chewing tobacco, and of these, 92% were under 25 years of age, precisely the demographic most rapidly adopting novel nicotine products. These findings are consistent with evidence that structured cessation support can be effective for smokeless tobacco users [55, 56] and underscore the importance of developing and scaling such programmes alongside efforts to generate better evidence on long‐term cardiovascular risk.

## Author contributions

**Johan Sundström**: Conceptualization; writing—review and editing; methodology. **Magnus Olsson**: Conceptualization; investigation; writing—original draft; methodology; visualization; writing—review and editing; project administration; software; formal analysis; data curation. **Michael Fored**: Conceptualization; writing—review and editing; methodology. **Daniel Jönsson**: Conceptualization; methodology; writing—review and editing. **Eva Nordendahl**: Conceptualization; writing—review and editing; methodology. **Björn Klinge**: Conceptualization; writing—review and editing; methodology. **Peter Persson**: Conceptualization; methodology; writing—review and editing. **Jakob Hedberg**: Conceptualization; writing—review and editing; methodology. **Aron Naimi‐akbar**: Conceptualization; methodology; project administration; supervision; resources; funding acquisition; validation. **Hanne Tønnesen**: Conceptualization; supervision; methodology; investigation; writing—review and editing; validation. **Cecilia Magnusson**: Conceptualization; writing—review and editing; methodology.

## Funding information

This study was supported by the Knowledge Foundation grant number 20250008 to the Advancing Oral Health research profile

## Conflict of interest statement

The authors declare no conflicts of interest.

## Data Availability

Data sharing is not applicable to this article, as no datasets were generated or analysed during the current study.
